# *Clostridium difficile*–related Hospitalizations among US Adults, 2006

**DOI:** 10.3201/eid1501.080793

**Published:** 2009-01

**Authors:** Marya D. Zilberberg

**Affiliations:** University of Massachusetts, Amherst, Massachusetts, USA; Evi*Med* Research Group, LLC, Goshen, Massachusetts, USA

**Keywords:** Clostridium difficile-associated disease, hospital, population epidemiology, United States, letter

**To the Editor:** The threat to public health posed by *Clostridium difficile*-associated disease (CDAD) continues to increase within and outside the United States. In a recent analysis, we detected a 23% annual increase in CDAD-related hospitalizations from 2000 through 2005 and a near-doubling in the associated age-adjusted case-fatality rate from 2000 through 2004 (*1*). In view of the aging US population, this rapid growth, along with the increased virulence and diminished susceptibility to antimicrobial drug treatments, if sustained, will not only strain the US healthcare system (*2*,*3*) but also will cause significant illness and death. For this reason, understanding up-to-date trends in CDAD-related hospitalizations is critical. Since the Agency for Healthcare Research and Quality recently made available its 2006 update to the National Inpatient Sample (NIS) data (*4*) on the Healthcare Costs and Utilization Project Net (HCUPNet) website (*5*), I explored the trends in CDAD hospitalizations beyond our 2005 estimates.

From the HCUPNet website (*5*), I identified CDAD-related hospitalizations for 2000–2006 in the NIS data (*4*). The NIS is a stratified 20% sample of US community hospitals, and data are weighted to provide national estimates (*4*). CDAD was identified by presence of the International Classification of Diseases, 9th revision, Clinical Modification (ICD-9-CM) diagnosis code 8.45 (intestinal infection with *Clostridium difficile*), and the numbers of discharges per year were age-stratified. To benchmark CDAD incidence against the general growth in hospitalizations over time, I obtained age-stratified numbers of total hospitalizations from HCUPNet for each year and derived CDAD-related hospitalization incidence as a function of total annual US hospitalization volume. I also obtained censal and intercensal data on the numbers and demographic characteristics of the US population during 2000–2006 from the US Census Bureau (*6*). On the basis of these data, I calculated age-specific hospitalization incidence rates and repeated these analyses by census region (Northeast, Midwest, South, and West). Finally, I explored trends in CDAD principal diagnosis hospitalizations as a proportion of all CDAD-related hospitalizations.

Overall, the rate of growth in CDAD-related hospitalizations slowed in 2006, with a crude volume increase of 6.7% over 2005; the difference in the raw volume from 2005 through 2006 did not reach statistical significance (p = 0.33). When hospitalization incidence was stratified by age, the greatest increase from 2005 through 2006 occurred in the 45- to 64-year age group (7.2%, from 8.48 to 9.09 cases per 10,000 population) and the smallest increase occurred in the >85-year group (0.9%, from 110.71 to 111.70 per 10,000 population); the 18- to 44-year (5.7%, from 2.26 to 3.39 per 10,000 population) and 65- to 84-year groups (5.1%, from 46.57 to 48.96 per 10,000 population) had results between the other 2 groups (Table). Although consistently higher in women than men, the population incidence of CDAD-related hospitalizations in men (4.0%) and women (5.0%) rose similarly (Table). In addition, although the volume of CDAD principal diagnosis had not previously exceeded 25% of total CDAD-related hospitalizations (*1*), in 2006 this proportion increased to 28% (Table). In examining CDAD hospitalization trends regionally, in the Northeast I found a directional reduction in incidence at both the total hospitalizations level and population level. CDAD-related hospitalizations in the Midwest, South, and West continued to increase (Table).

**Table:** Incidence of *Clostridium difficile*–related hospitalizations among US adults, 2000–2006, by age group and region*

Hospitalization variable	Year						
	2000	2001	2002	2003	2004	2005	2006
All US adults per 10,000 population							
18–44 y	1.31	1.33	1.66	1.71	1.96	2.26	2.39
45–64 y	4.53	4.58	5.92	6.31	7.20	8.48	9.09
65–84 y	22.41	23.94	31.61	33.74	39.06	46.57	48.96
>85 y	52.09	57.03	70.15	74.99	89.39	110.71	111.70
Principal diagnosis CDAD as a proportion of total CDAD, %							
18–44 y	26.3	26.8	26.9	26.1	28.1	29.1	30.7
45–64 y	24.4	25.2	24.4	23.2	24.3	25.5	27.4
65–84 y	22.8	23.7	23.3	22.8	23.8	24.9	27.8
>85 y	22.1	24.4	23.6	23.2	24.0	25.0	29.8
By sex per 10,000 population							
M	4.10	6.30	5.71	6.03	7.03	8.59	9.00
F	5.72	6.83	7.71	8.32	9.71	11.72	12.29
By sex per 1,000 hospitalizations							
M	3.83	5.81	5.24	5.54	6.43	7.79	8.13
F	3.81	4.51	5.04	5.45	6.36	7.66	8.02
Regionally per 10,000 population							
Northeast	7.05	7.59	9.30	10.40	10.73	14.46	14.28
Midwest	5.70	5.32	7.96	8.51	9.86	11.32	12.27
South	4.05	4.98	6.26	6.49	8.19	9.50	9.64
West	3.72	3.52	4.10	4.39	5.39	6.73	7.32
Regionally per 1,000 hospitalizations							
Northeast	5.15	5.52	6.74	7.47	7.64	10.17	10.00
Midwest	4.36	3.98	5.90	6.31	7.26	8.26	8.97
South	2.97	3.59	4.49	4.66	5.89	6.83	7.06
West	3.41	3.24	3.76	4.00	4.92	6.16	6.85

*CDAD, *C. difficile*–associated disease.

While previously growing at the unsustainable rate of 23% per year, from 2005 through 2006, CDAD-related hospitalizations in adults increased 6.7%, representing a potential considerable slowing of the epidemic. Whereas the number and incidence of CDAD-related hospitalizations decreased in the Northeast, such hospitalizations in the remaining regions had sustained, albeit smaller than in prior years, increases. Additionally, the proportion of all CDAD-related hospitalizations for which CDAD was the principal diagnosis for the hospitalization rose to 28% overall. Given the definition of a “principal diagnosis” in the NIS database as “that condition established after study to be chiefly responsible for occasioning the admission of the patient to the hospital for care; the principal diagnosis is always the reason for admission,” this rise could represent altered disease manifestation, severity, or virulence.

In summary, the previously noted rate of growth in CDAD-related hospitalizations in US adults appears to have slowed somewhat in 2006. The encouraging downward trend in CDAD in the Northeast requires further exploration. Of concern is the increasing proportion of CDAD-related hospitalizations coded as the primary reason for admission because this may signal a change in characteristics of the disease. Given that the new data represent only 1 year, and the difference between the raw numbers from 2005 through 2006 did not reach statistical significance, these findings need to be interpreted with caution and monitored annually. In general, although helpful, most nationwide data have a considerable lag time. In this and other resistant diseases sweeping the US hospitals, real-time surveillance data are needed for more prompt and actionable policy development.
